# Virtual Training of the Myosignal

**DOI:** 10.1371/journal.pone.0137161

**Published:** 2015-09-09

**Authors:** Bernhard Terlaak, Hanneke Bouwsema, Corry K. van der Sluis, Raoul M. Bongers

**Affiliations:** 1 University of Groningen, University Medical Center Groningen, Center for Human Movement Sciences, Groningen, the Netherlands; 2 Centre of Expertise in Rehabilitation and Audiology, Adelante Rehabilitation Centre, Hoensbroek, The Netherlands; 3 Department of Rehabilitation Medicine, Maastricht University, Research School CAPHRI, Maastricht, The Netherlands; 4 University of Groningen, University Medical Center Groningen, Department of Rehabilitation Medicine, Groningen, the Netherlands; Duke University, UNITED STATES

## Abstract

**Objective:**

To investigate which of three virtual training methods produces the largest learning effects on discrete and continuous myocontrol. The secondary objective was to examine the relation between myocontrol and manual motor control tests.

**Design:**

A cohort analytic study.

**Setting:**

University laboratory.

**Participants:**

3 groups of 12 able-bodied participants (N = 36).

**Interventions:**

Participants trained the control over their myosignals on 3 consecutive days. Training was done with either myosignal feedback on a computer screen, a virtual myoelectric prosthetic hand or a computer game. Participants performed 2 myocontrol tests and 2 manual motor control tests before the first and after the last training session. They were asked to open and close a virtual prosthetic hand on 3 different velocities as a discrete myocontrol test and followed a line with their myosignals for 30 seconds as a continuous myocontrol test. The motor control tests were a pegboard and grip-force test.

**Main Outcome Measures:**

Discrete myocontrol test: mean velocities. Continuous myocontrol test: error and error SD. Pegboard test: time to complete. Grip-force test: produced forces.

**Results:**

No differences in learning effects on myocontrol were found for the different virtual training methods. Discrete myocontrol ability did not significantly improve as a result of training. Continuous myocontrol ability improved significantly as a result of training, both on average control and variability. All correlations between the motor control and myocontrol test outcome measures were below .50.

**Conclusions:**

Three different virtual training methods showed comparable results when learning myocontrol. Continuous myocontrol was improved by training while discrete myocontrol was not. Myocontrol ability could not be predicted by the manual motor control tests.

## Introduction

Myocontrol is the control of an external device through electromyography (EMG) signals derived from the action potentials produced by the muscles (the myosignals) and is used in myoelectric prostheses. In conventional myoelectric prostheses the myosignals of two muscles are used as input to control opening and closing functions of a prosthetic hand. Importantly, recently developed multi-articulating prosthetic hands have the possibility of using more grip patterns [1–4], while the number of control signals has not changed. To perform different grip types more and more detailed properties of the myosignal need to be controlled, such as proportional control and co-contraction [5,6]. Producing such advanced patterns of the myosignals requires dexterous myocontrol, putting high demands on the training of an amputee’s myocontrol and also asking much from the amputee’s learning abilities.

Knowledge about characteristics of training that improve myocontrol might therefore be helpful for both clinicians and amputees. Such knowledge may increase the amputee’s ability to control his myosignals, which affects his abilities with a prosthetic hand. As previous research has shown [7–12], myocontrol can be improved by training. Furthermore, virtual training methods have also proven to be effective [9–12]. Interestingly, to our knowledge earlier research never compared different virtual training methods. A possible advantage of virtual training is that it can start much earlier after amputation than conventional training, as a fully healed stump is not required. As a consequence, neuroplasticity processes at work after amputation can be optimally utilized (for an overview of these processes see Di Pino et al. [13]). Such an early start of training is in line with the concept that starting training early (generally within 30 days) after amputation leads to higher prosthetic use [14–17]. In addition, virtual training is very cost effective as less expensive hardware is required (i.e., fitting of a prosthesis is not required) and off the shelf computer games might be used for training.

Virtual training methods for prosthesis myocontrol come in three broad classes. Firstly, a basic class, in which control of the myosignal is trained by displaying the signals as feedback on a computer screen. A second class of training methods presents a virtual prosthesis on a screen where the control is identical to that of an actual prosthesis [18,19]. Lastly, some computer games for training purposes incorporate myosignals to control specific features of the game [20,21]. In the current study, these three classes of myocontrol training will be tested.

To reveal which of these training types has the highest effect on myocontrol performance two aspects of myocontrol will be tested: discrete myocontrol, implying short activations that can vary in intensity, and continuous myocontrol, implying prolonged myosignal activity with continuous intensity changes. Each myocontrol type is used in modern myoelectric prostheses, for example in grasping (discrete) and continuous adjustments or grip switching in more complex tasks (continuous). Both forms of myocontrol are thus needed to accurately control a prosthetic hand.

Besides knowledge about characteristics of training to improve myocontrol it would also be beneficial for the clinical setting to be able to determine an amputee’s myocontrol learning ability in a simple low-tech way before acquiring a prosthesis. In a previous study, Bouwsema et al. [11] found large individual differences in the ability to produce distinct myosignals. In their experiment, participants trained myocontrol and were tested on opening and closing a prosthetic hand at 3 different velocities. Based on the regression slope calculated over the opening and closing velocities, participants were classed as either high-capacity learners (HCL) or low-capacity learners (LCL). Analyses showed that HCL could produce significantly more distinct myosignals. This finding is important for the clinical setting as an amputee’s myocontrol ability is directly related to their ability to use the proportional control of a prosthetic hand. Virtual training software offers the tools to determine a patient’s myocontrol ability. A more low-tech way to establish learning ability, that might be preferable in the clinical setting, would be to measure manual, gross and fine motor control in simple tests. However, using these tests to establish myocontrol ability is only valid if a relation exists between the scores on motor control tests and myocontrol ability. Obviously, the skills tested in motor control tests are different from myocontrol ability. The current study intends to reveal whether these skills are related; although manual tests and myocontrol tests reflect different skills, the scores on the manual tests and the myocontrol tests might relate when there is something like a general motor skill. Moreover, it might be the case that these skills relate differently for the dominant and the non-dominant hand.

This study therefore has two aims: 1) Examine which virtual training method has the largest effect on learning discrete and continuous myocontrol. 2) Examine whether there is a relation between myocontrol and manual motor control tests. We hypothesized that virtual training with a computer game will result in larger learning effects than training with myosignal feedback or a virtual myoelectric hand, both on discrete and continuous myocontrol, because research has shown that a virtual gaming environment results in higher training motivation [22–24].

## Methods

Thirty-six able-bodied right-handed participants were studied: 18 men (mean age 22.8y) and 18 women (mean age 22.2y). The size of the sample was based on the data of Bouwsema et al. 2010 [11]. Using the Morepower software [25] we computed the number of participants required using an alpha of 0.05 and a power of 0.80. Inclusion criteria were as follows: normal or corrected to normal eye-sight, right-handed, free of any neurological or musculoskeletal problems concerning the upper extremity or torso and no prior experience with prosthetic myocontrol. The study was approved by the Medical Ethical Committee of the University Medical Center Groningen, the Netherlands (METc UMCG, NL39792.042.12). Written informed consent was given by each participant before the start of the experiment.

### Design

Using a computer program, participants were randomly assigned to one of three training groups, with the restriction that there was an equal distribution of males and females over the three groups. Each of these groups trained with a method included in the Ottobock PAULA^a^ (Prosthetists’ Assistant for Upper Limb Architecture) software. The first group (Myo) trained with their myosignals as feedback displayed on a computer screen. The second group (VH) trained with a virtual myoelectric prosthetic hand presented on a screen controlled in the same way as an actual prosthesis, and the third group (Game) trained with a computer game in which they controlled two cars through myocontrol. Because amputations can occur on both sides, one half of the participants trained with their dominant side and the other half with their non-dominant side. The same computer program that did the randomisation also assigned the hand with which each participant trained. The experiment was conducted on three consecutive days. On the first day, the participants performed two manual motor control tests, followed by a pre-test to determine myocontrol baseline skills. Subsequently, myocontrol was trained on three consecutive days. Each of the groups trained 6 sessions of 2 minutes each day, with a 30 second break between each session. After training on the third day, the same tests as in the pre-test were administered as a post-test followed by the two manual motor control tests.

### Materials and procedures

#### Pre-test and post-test: manual motor control tests

The pegboard task from the movement ABC2 (mABC2, Henderson SE, Sugden DA, Barnett AL. *Movement Assessment Battery for Childeren– 2 Examiner’s Manual*. London: Harcourt Assessment, 2007), to assess fine motor control, and a test of grip force control using a grip force dynamometer to assess gross motor control, were used to determine the level of motor control of the participants. Participants performed each test twice with their normal hands, once with the dominant and once with the non-dominant hand, the order of which was balanced over participants. Execution of the pegboard task followed instructions from the mABC2. Grip force measurements were based on the standard protocol for a Jamar hand-held dynamometer and modified to ask the production of 25%, 50%, 75% and 100% of maximum grip force.

#### PAULA: electrode placement and calibration

Ottobock PAULA^a^ software, in conjunction with 75M11Myoboy with active socket electrodes (13E200 Myobock electrodes with a rectified and filtered [2^nd^ order] output and a linear sensitivity controller) connected through USB to a pc, was used for training and electrode placement. One electrode was placed on the wrist flexor muscle and one other electrode was placed on the wrist extensor muscle. The procedure of electrode placement was the same as done previously [11,26]: The exact positions of the electrodes were determined after palpation of the most prominent contraction of the muscle bellies of the extensors and flexors of the wrist. The sensitivity of the electrodes was adjusted to the upper threshold—a high level of myoelectric signal—for each participant individually. This fitting procedure had to be repeated each day before the start of the training to prevent environmental factors, such as perspiration of the skin, influence the myoelectric signals that were picked up by the electrodes. The locations of the electrodes were marked. This location was taken as the starting point for the procedure to place the electrodes described in the above so that the electrodes could be placed at approximately the same position every experimental day.

#### Pre-test and post-test: myocontrol measurements

Custom software was developed to test discrete and continuous myocontrol abilities in separate tests. Both tests were applied to both arms of each participant. The order of measuring the arms was balanced over participants. The tests were taken from both hands because we aimed to examine whether training with the one side would transfer to the other side [26, 27]. This would allow to start training with a virtual training tool immediately after an amputation.

The test of discrete myocontrol used a virtual prosthetic hand with proportional control (higher myosignal activity leading to faster movements). Participants had to open and close the virtual prosthetic hand to full aperture at either the slowest, moderate or fastest controllable velocity per trial (cf. Bouwsema et al. [11]). All velocities were executed three times in a random order (total of 9 trials). Myosignal levels and hand speed over time were sampled at 100Hz.

**Fig 1:**
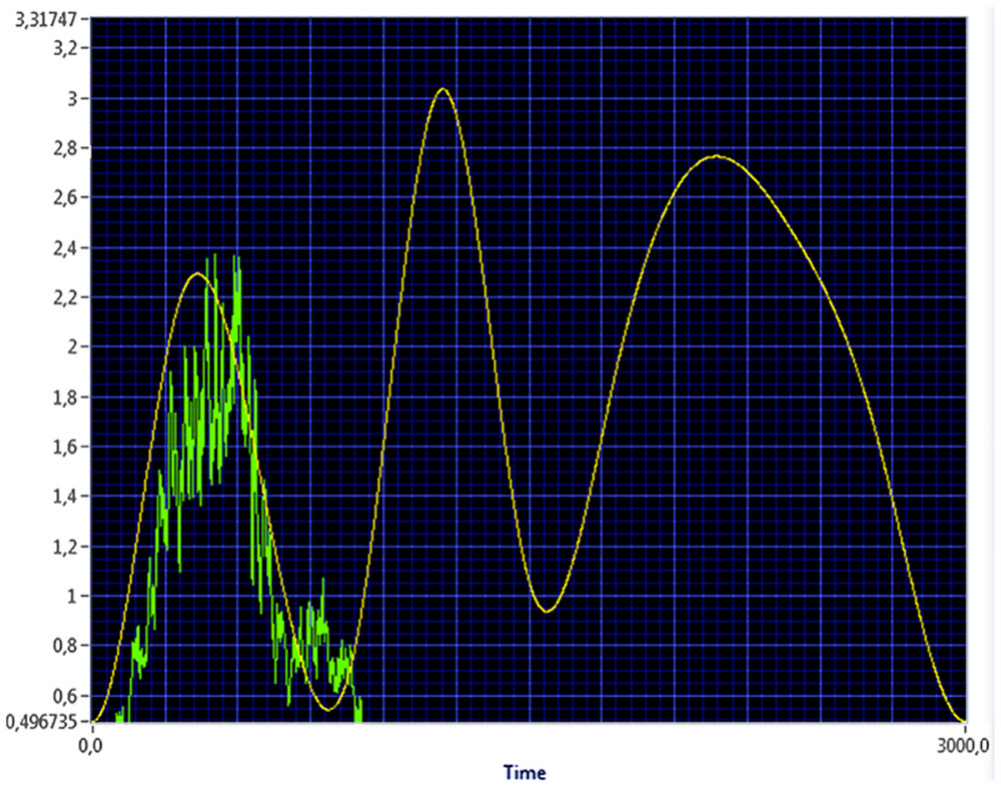
Continuous myocontrol task. Over a period of 30 second, a semi-random line, based on the participant’s myosignal range, had to be followed as closely as possible with either the wrist flexor or extensor myosignals. The figure contains an example of the task being performed with the flexor myosignals. X-axis: time, 30 seconds with 100Hz measurements. Y-axis: myosignal range in Volts.

Testing of continuous myocontrol used myosignal feedback and a semi-random graph to which the participants had to match their myosignals over a 30 second period (Fig 1). This graph consisted of the 10^th^ to 90^th^ percentile (in steps of 10%) of the participant’s myosignal range, placed in a random order and combined through a spline function. The test was performed twice, with either the flexor or extensor muscles, used to control closing and opening of the hand respectively.

#### Training sessions

PAULA software (757M11 Myoboy; 13E200 MyoBock Electrodes; Otto Bock HelathCare Products Ges mbH, Vienna, Austria) was also used for the training, as it contained direct myosignal feedback training, a virtual myoelectric prosthetic hand and a computer game training mode. Participants of all groups were instructed that they were meant to consciously influence and improve the control over their myosignals. With this instruction we aimed to focus the participants on learning the relation between the produced muscle contraction and an effect on the screen, specified either as myosignals, a virtual hand, or the avatar in a game. During the training an experimenter was always present. The experimenter stimulated participants to explore the whole range of the myosignal. In this way we aimed to match the effort and explored range of the myosignal over all the training groups.

**Fig 2:**
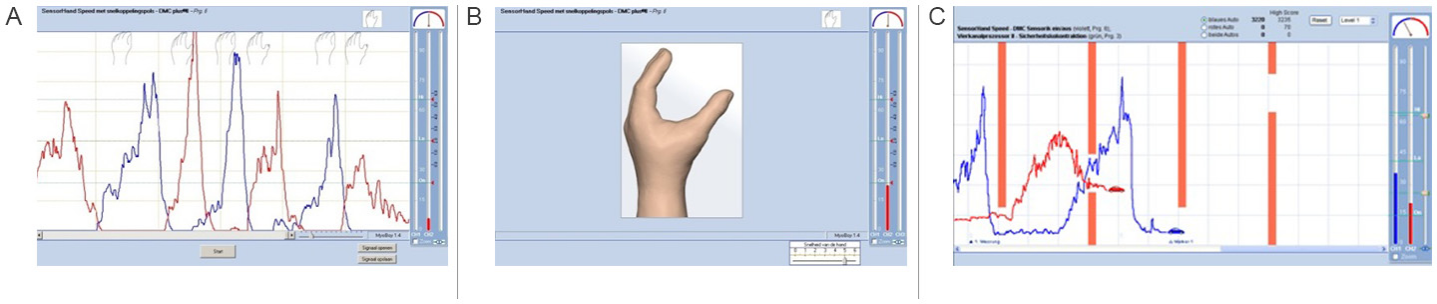
Myosignal training modes. (a) Myosignal feedback training mode, (b) virtual prosthetic hand training mode, and (c) the computer game training mode.

The Myo group, training with their produced myosignals as instantaneous feedback (Fig 2a) was instructed that stronger contraction would lead to higher signals and were told to train 2 minutes per round. The signals of both muscles were presented together on the screen. Participants were instructed to explore the range of the myosignal and pay attention to the relation between variations in muscular effort and the results in the signals. The VH group, training with a virtual myoelectric prosthetic hand (Fig 2b), was instructed that flexor and extensor contractions led to closing and opening of the hand on the screen and stronger contractions led to faster movements. They were told to train 2 minutes per round without further specific instructions on the exact movements to make. The Game group had to control the vertical movements of two cars with their myosignals (each muscle group controlling movements of one car), and steer these through gaps in oncoming walls by producing myosignals of the correct height (Fig 2c) for 2 minutes per round. One car had to be steered through the gap per wall, switching cars each wall.

### Data analysis

Custom-written scripts in Matlab (The MathWorks, Natick, MA, USA) were used to compute the following dependent variables: mean velocity of the hand opening and closing for each required velocity (slow, moderate, fast) for the discrete test; the error between the produced myosignals and the predefined graph, and error SD for the continuous test. The error was averaged over the trial and normalised by dividing it by the participant’s myosignal range.

Table 1 contains the study design, outcome variables and the performed analyses on the outcome variables. In case of the ANOVA’s, when sphericity was violated, the degrees of freedom were adjusted with the Greenhouse-Geisser correction. In all analyses, a significance criterion of α < .05 was used, and post-hoc tests used Bonferroni corrections. Generalized eta squared [28,29] was used to calculate effect sizes, which were interpreted according to Cohen’s recommendations [30] of .02 for a small effect, .13 for a medium effect and .26 for a large effect.

**Table 1:** Study design, outcome measures and data-analyses.

Day	Program	Tasks	Outcome measures	Analyses
1	Pre-test: motor control tests	Pegboard task	Test completion time	1,2
		Grip-force task	Regression slope over the grip force scores.	1,2
	Pre-test: myocontrol tests	Discrete myocontrol test	Mean velocity per condition (slow, moderate, fast)	3
			Regression slope over the 3 mean velocities	1,2
		Continuous myocontrol test	Error	1,2,4
			Error SD	1,2,5
	Training*	6 x 2 minutes effective training time	-	
2	Training*	6 x 2 minutes effective training time	-	
3	Training*	6 x 2 minutes effective training time	-	
	Post-test: myocontrol tests	Discrete myocontrol test	Mean velocity per speed (slow, moderate, fast)	3
			Regression slope over the 3 mean velocities	2
		Continuous myocontrol test	Error	2,4
			Error SD	2,5
	Post-test: motor control tests	Pegboard task	Test completion time	
		Grip-force task	Regression slope over the grip force scores.	

* Training differed between the 3 groups and was either myosignal feedback training, virtual myoelectric prosthetic hand training or computer game training.

Analyses:

1. Pearson correlations between the motor control pre-test scores and the myocontrol post-test scores: examining the predictive value of the motor control tests on myocontrol ability after training.

2. Pearson correlations between the motor control pre-test scores and the myocontrol pre-to-post-test difference score: examining the predictive value of the motor control tests on myocontrol learning ability.

3. Repeated Measures ANOVA: dependent variable: discrete test mean hand velocity; within subject factors: test (pre-test, post-test), tested hand (right, left), velocity (slow, moderate, fast); between subject factor: group (Myo, VH, Game).

4. Repeated Measures ANOVA: dependent variable: continuous test error; within subject factors: test (pre-test, post-test), tested hand (right, left), muscle (extensor, flexor); between subject factor: group (Myo, VH, Game).

5. Repeated Measures ANOVA: dependent variable: continuous test error SD; within subject factors: test (pre-test, post-test), tested hand (right, left), muscle (extensor, flexor); between subject factor: group (Myo, VH, Game).

## Results

The data can be found in the Supporting Information files. Note that in each group half of the participants *trained* with the dominant side and the other half trained with the non-dominant side Explorative data analyses showed only one effect with effect size less than .02 on dominance of *training hand* and one effect with effect size less than .02 on gender, in both myocontrol tests. Additionally, no effect of direction (opening, closing) was found in the discrete test. Consequently, these factors (training hand, gender, and direction of hand opening) were not further analysed. Note, moreover, all participants performed the tests with both hands. The factor ‘test hand’ was included in the analyses (see Table 1).

### Training myocontrol

#### Discrete myocontrol

Training groups did not differ significantly in their learning effect on discrete myocontrol (Table 1, analysis 3. As expected, a large effect of velocity was found F_(2.60)_ = 562.42, p = .00, η^2^_g_ = .68. In the fast condition, the participants reached the highest velocities (500.23 mm/s (11.32) (mean[SE])), whereas the lowest were reached in the slow condition (186.82 mm/s (8.03)). No significant effect of test or any significant interaction was found.

#### Continuous myocontrol–error

**Fig 3:**
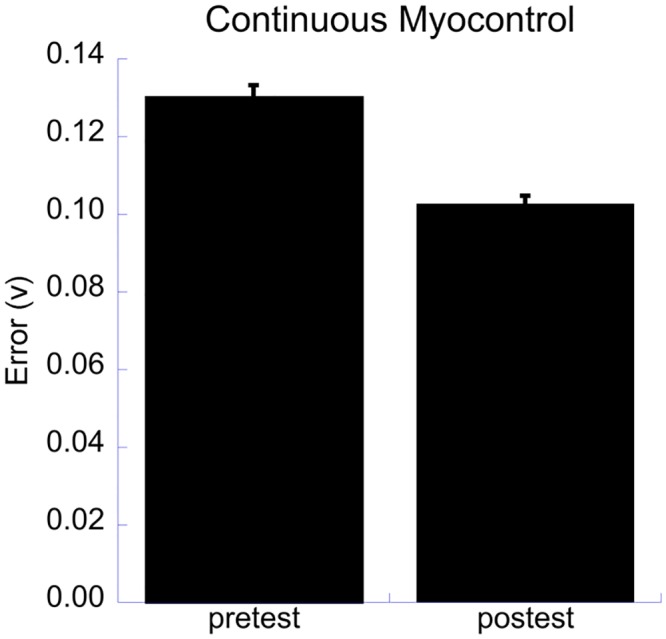
Error in the continuous myocontrol test. The significant main effect of test for the error in the continuous myocontrol test.

No significant differences between the learning effects of the three training groups were found on the error (Table 1, analysis 4). The main effect of test had a medium strength and was the largest effect size found (Table 2), participants improved significantly from pre-test to post-test (Fig 3). Additionally, small effects of muscle, test x muscle and tested hand x test x group were found (Table 2). Post hoc analysis on the latter effect showed that the Myo-group progressed more on the test performance with their right hand than the Game-group (Myo-group: pre-test .1425 (.0063), post-test .1024 (.0037); Game-group: pre-test .1219 (.0057), post-test .0997 (.0041)).

**Table 2:** Overview of significant effects on the continuous myocontrol test.

Dependent variable	Main effect or interaction effect	F	P	η _g_^2^
Error	Test	87.13	.00	.20
	Tested hand x test x group	8.80	.001	.04
	Muscle	16.70	.00	.06
	Test x muscle	8.87	.006	.02
Error SD	Test	58.86	.00	.15
	Tested hand x test x group	14.78	.00	.06
	Muscle	22.97	.00	.07

Error df was 30 in all cases. Effect df was 1 in the effects not involving ‘group’, otherwise the df was 2. Repeated Measures ANOVA factors: within subject factors: test (pre-test, post-test), tested hand (left, right), muscle (extensor, flexor); between subject factor: group (Myo, VH, Game).

#### Continuous myocontrol—error SD

**Fig 4:**
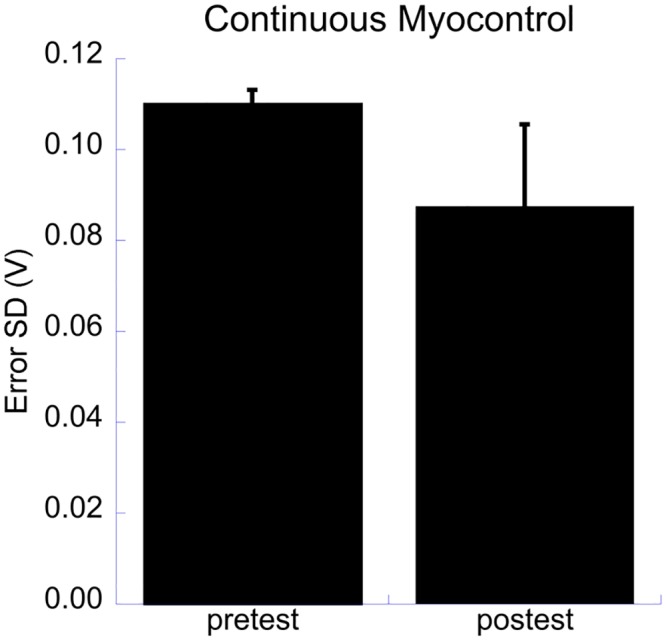
Standard deviation of the error in the continuous myocontrol test. The significant main effect of test for the error SD in the continuous myocontrol test.

Training groups did not differ in their learning effects on the SD of the error (Table 1, analysis 5). The largest effect size found was the medium main effect of test (Table 2), participants produced more stable signals from pre-test to post-test (Fig 4). Additionally, small effects of muscle and tested hand x test x group were found (Table 2). Post hoc analysis of the latter effect showed that the Myo-group progressed more on the test performance with their right hand than the Game-group (Myo-group: pre-test .1276 (.0077), post-test .0846 (.0032); Game-group: pre-test .1001 (.0053), post-test .0836 (.0038)).

### Manual motor control tests and myocontrol

#### Manual motor control tests

Table 3 presents an overview of the scores on the manual motor control tests. This overview shows that the groups differed somewhat in their performance on these tests but that these differences were not substantial.

**Table 3:** Overview of the pre-test scores of the manual control tests. SEM is Standard error of the mean, Pin is the pegboard test score and Grip is the grip force test, D is dominant and ND is non-dominant.

			Test			
Group	TrainingHand	Variable	PinD	PinND	GripD	GripND
Car	Dominant/Right	Mean	19.30	21.35	15.65	13.68
		SEM	1.87	1.43	1.79	4.18
	Non-Dominant/Left	Mean	18.15	22.03	17.94	16.23
		SEM	1.70	1.40	2.37	2.48
Myo	Dominant/Right	Mean	20.48	22.05	19.31	20.49
		SEM	0.72	0.71	3.38	3.60
	Non-Dominant/Left	Mean	17.35	20.78	19.07	16.87
		SEM	0.87	0.98	3.62	1.57
VH	Dominant/Right	Mean	17.13	21.78	15.22	20.92
		SEM	1.00	0.99	2.73	4.01
	Non-Dominant/Left	Mean	17.37	18.13	12.80	18.41
		SEM	0.52	0.84	2.74	3.58
SemiTotal	Dominant/Right	Mean	18.97	21.73	16.73	18.36
	Non-Dominant/Left	Mean	17.62	20.32	16.60	17.17
Total	Total	Grand Mean	18.30	21.02	16.67	17.77

#### Correlation manual motor tests and mycontrol

All correlations between the manual motor control test scores and the discrete and continuous myocontrol test scores (Table 1, analysis 1) were lower than .50 (Table 4). This was also true for the correlations between the motor control test scores and the participant’s learning curves (Table 1, analysis 2) on both myocontrol tests (Table 4). These low correlations showed that the scores on the manual motor control tests were not related to measures of discrete and continuous myocontrol.

**Table 4:** Pearson correlations between the manual motor control pre-test scores and the discrete and continuous post-test scores (myocontrol ability) and the difference score from pre-test to post-test (myocontrol learning ability) on the discrete and continuous tests.

		Myocontrol ability						Myocontrol learning ability					
		Cont: Error PostD	Cont: Error PostND	Cont: ErrorSD PostD	Cont: ErrorSD PostND	Disc: PostD	Disc: PostND	Cont: Error PrePostD	Cont: Error PrePostND	Cont: ErrorSD PrePostD	Cont: ErrorSD PrePostND	Disc: PrePostD	Disc: PrepostND
PegPreD	r	-.046	.096	.094	.083	-.148	.270	-.130	.014	-.066	.064	-.093	-.018
	p	.778	.578	.586	.631	.389	.111	.451	.934	.701	.710	.589	.915
PegPreND	r	-.304	.014	-.296	-.008	-.088	-.011	-.370	-.040	-.260	.001	-.106	-.123
	p	.072	.937	.080	.964	.610	.950	.026*	.819	.126	.997	.539	.476
GripD	r	-.268	-.003	-.323	.064	.169	-.152	-.176	.135	-.201	.201	.055	-.078
	p	.113	.988	.055	.710	.324	.376	.306	.433	.239	.239	.750	.650
GripND	r	-.024	-.184	-.018	-.102	.160	.018	.237	.104	.088	.078	.061	.061
	p	.889	.281	.916	.555	.353	.917	.164	.546	.610	.649	.722	.726

* Indicates a significant difference at the .05 level.

D = Dominant hand test scores. ND = Non-Dominant hand test scores. Pre = Pre-test scores. Post = Post-test scores. PrePost = difference in scores from pre- to post-test. Peg = Pegboard task. Grip = Regression slope on the grip-force task scores. Cont = Continuous test variables. Error = mean normalised error between the line and the produced myosignals. ErrorSD = standard deviation of the error. Discrete = Discrete test variables, calculated as the regression slopes over the three velocities.

## Discussion

The current study showed that virtual training of myoelectric control using direct feedback of the produced myosignals projected on a computer screen, a virtual prosthetic hand, or a computer game does not differ for discrete and continuous aspects of myocontrol. As such, in the clinical practice the available method can be used based on the needs and preferences of the patient. We hypothesized that the computer game would produce larger learning effects as a result of higher evoked training motivation. A possible explanation for the fact that we had to reject our hypothesis might be that the duration of the training was too short. Effectively, participants trained for 36 minutes over 3 days, which may be too short to take advantage of the benefits of training with a computer game. As literature was unable to provide adequate information on the required training time for an optimum in duration of training, we based the current design on our earlier study (Bouwsema et al. [11]). Note, however, that our choice of the length of the training was informally substantiated because in the last session several participants mentioned that they started to get bored by the training. This might indicate that the game was not sufficiently engaging enough or learning started to plateau. From the results it became clear that participants significantly improved their continuous myocontrol ability through training, while they did not significantly improve their discrete myocontrol. These findings imply that continuous myocontrol can be learned through virtual training. Additionally, it shows that discrete myocontrol may be harder to learn and shows the necessity for more knowledge regarding the effects of different training methods, feedback and duration, both per session and number of sessions.

Interestingly, the correlations between the pegboard and grip force pre-test scores and the post-test scores on the discrete (regression slopes on the 3 velocities) and continuous (error and error SD) myocontrol tests showed that these tasks were incapable of predicting myocontrol ability. Additionally, the correlations between the motor control pre-test scores and the difference on the myocontrol pre- to post-test scores showed that myocontrol learning ability could not be predicted by the motor control tests either. These findings demonstrate that the myocontrol performance does not seem to be related to manual motor control tests. This may indicate that although myosignals produce motor behaviour required to execute both the motor control tests as well as the signals controlling the virtual trainings and myocontrol tests, training myocontrol does not affect all motor behaviours. However, as only two motor control tests were performed, different tests might provide other results.

We recommend that future research should try to find or construct an easily administrable test to identify myocontrol learning ability. Our previous research showed that there are large individual differences in the ability to produce distinct myosignals [11]. As this ability is strongly related to the prosthetic functions that can be adequately controlled, having an easily administrable test to identify myocontrol learning ability could help in selecting a prosthetic device fitting to the abilities of an amputee. As myocontrol is a complex skill, incorporating the control of a prosthetic device in a motor control tests could lead to insightful results. A task based on wrist flexion and extension could therefore be a viable option.

We showed that participants can learn discrete and continuous myocontrol using a training in different virtual tasks. To establish this learning we used tests that were different from the training tasks. To what extent does this learning depend on the tasks that are used or the mapping between myosignal and what happens on the screen? Other studies demonstrating effects of learning myocontrol often do not have separate test tasks [31–35]. These studies also showed learning effects that did not depend on the training. For instance, Pistohl et al 2014 found only marginal differences in learning to control a cursor in a reach center-out task and controlling different grips in a multi-articulated table-top hand, using four EMG electrodes attached to hand muscles, over the stages of learning. Our control scheme was less complicated but we also found no statistical differences between the virtual hand training group and the other two groups when comparing pre-test with post-test. Moreover, the group who had to produce myosignals on the screen had not learned the specifics of the mapping between a myosignal and the opening of the virtual hand or the movement of a virtual car in a game. Still, these latter two groups did not perform better in the discrete myocontrol post-test in which the mapping was incorporated. That participants have the ability to use their skills of controlling the myosignal in a task with another mapping is in agreement with the findings of Antuvan et al. [31] who found that skills in one mapping could transfer to another mapping between four EMG electrodes on arm muscles and a two degree of freedom task.

### Study limitations and recommendations for future research

Able-bodied participants were used in the experiment rather than patients with a recent amputation, as there are very few recently amputated patients. By using able-bodied participants, we could measure a larger number of participants, resulting in more reliable measurements and results. Note however, that although we used results of another study [11] to perform power analyses, the participants of that study might have differed that much from the current participants that we would have found effects of training if we had used more participants. Moreover, future studies should use a set-up and a design that allowed examining the learning rate within each training, because our training groups might have differed in that respect.

A disadvantage of using able-bodied participants lies in the absence of neuroplasticity processes at work following in amputation (see Di Pino et al. [13]). Although a previous study by Schabowsky et al. [17] showed that the motor performance and learning ability of amputees were similar to those of unimpaired participants, caution should be taken when generalizing the current findings to amputees.

The current study tested the progress on myocontrol ability as a result of virtual training. While it was shown that the training led to an improvement in continuous myocontrol, this was also measured virtually. Although Bouwsema et al. [11] showed that myocontrol with a prosthetic simulator can be improved by virtual training, the extent of this effect and the transfer to functional tasks with a prosthesis remain to be tested. Additionally, no control group was used in the current study. Even though myocontrol is a complex skill, unlikely to be learned adequately by testing alone, it is advisable for future studies to incorporate a control group.

We showed improvement in pre-test post-test comparisons on the myocontrol tests but we did not find differences between different training groups. In this final part of the discussion we discuss whether these findings might result from that our instructions during the training program were not that specific, or from the particulars of the cargame we used. First, we should discuss whether participants have been training what they ought to train during the training sessions. Following our instructions, in all training groups participants had to explore and employ a wide range of myosignal strengths. The same was asked from participants in the test phases. Therefore, we are confident that although the test phases differed from the trainings, we tested the trained behaviour. Note that the experimenter stimulated the participants to explore the range of myocontrol. With this stimulation we aimed to achieve that all participants explored the same range of the myosignal. Because this stimulation standardized the range of exploration over participants we did not expect it to have affected the results.

Second, we expected higher learning rates for the group training with the cargame due to the presumed higher motivation in playing this game. Although, participants were more motivated to play this game than the other trainings, also the participants in this game indicated the game started to bore them after a few sessions. Perhaps, more motivating games, such as a moycontrolled Guitar Hero-game [36] could have demonstrated a higher learning effect.

## Conclusions

Three different virtual training methods showed comparable results when learning myocontrol. Continuous myocontrol was significantly improved by the provided training while discrete myocontrol was not.

Manual motor control tests seem inadequate to predict individual myocontrol learning ability and myocontrol ability after training.

## Supporting Information

S1 Data Correlation data—Virtual training of the myosignal. (TXT)

S1 Read Me Read me of correlation data—Virtual training of the myosignal. (DOCX)
