# Supplementary material for: Virtual Training of the Myosignal
Source: PLoS One. 2015 Sep 9;10(9):e0137161. doi: 10.1371/journal.pone.0137161 (PMC4564211; doi:10.1371/journal.pone.0137161)
Supplement: S1 Read Me — (DOCX) [file pone.0137161.s002.docx]

**S1 Read me of correlation data ­— Virtual training of the myosignal**

This document contains a brief explanation of the data found in the data-file of the Supplement.

The data is used for the correlation analysis as described under the header ‘Manual motor control tests and myocontrol’, which is a subsection of the Results.

All variables in the data set are given identical names to those used in Table 3.

The first two columns contain the participant number and their training method (group). For the group column, Game refers to the training method with the controllable cars, Myo to the myosignal training method and VH to the virtual hand training method.

Additionally, the subscript of this table, containing the explanation of the variable names and their properties is also used in the data file. For easy reference, the subscript is as follows:

D=Dominant hand test scores. ND=Non-Dominant hand test scores. Pre=Pre-test scores. Post=Post-test scores. PrePost= difference in scores from pre- to post-test. Peg= Pegboard task. Grip=Regression slope on the grip-force task scores. Cont= Continuous test variables. Error=mean normalised error between the line and the produced myosignals. ErrorSD= standard deviation of the error. Discrete= Discrete test variables, calculated as the regression slopes over the three velocities.

Further explanation of the procedures and variables can be found in the manuscript.
